# Identification of Novel Low-Density Neutrophil Markers Through Unbiased High-Dimensional Flow Cytometry Screening in Non-Small Cell Lung Cancer Patients

**DOI:** 10.3389/fimmu.2021.703846

**Published:** 2021-08-13

**Authors:** Paulina Valadez-Cosmes, Kathrin Maitz, Oliver Kindler, Sofia Raftopoulou, Melanie Kienzl, Ana Santiso, Zala Nikita Mihalic, Luka Brcic, Jörg Lindenmann, Melanie Fediuk, Martin Pichler, Rudolf Schicho, A. McGarry Houghton, Akos Heinemann, Julia Kargl

**Affiliations:** ^1^Otto Loewi Research Center, Division of Pharmacology, Medical University of Graz, Graz, Austria; ^2^BioTechMed, Graz, Austria; ^3^Diagnostic and Research Institute of Pathology, Medical University of Graz, Graz, Austria; ^4^Division of Thoracic and Hyperbaric Surgery, Department of Surgery, Medical University of Graz, Graz, Austria; ^5^Division of Oncology, Department of Internal Medicine, Medical University of Graz, Graz, Austria; ^6^Clinical Research Division, Fred Hutchinson Cancer Research Center, Seattle, WA, United States; ^7^Human Biology Division, Fred Hutchinson Cancer Research Center, Seattle, WA, United States; ^8^Division of Pulmonary and Critical Care Medicine, University of Washington, Seattle, WA, United States

**Keywords:** low-density neutrophils, non-small cell lung cancer (NSCLC), high-dimensional flow cytometry, CD36, CD41, CD61, CD226

## Abstract

Neutrophils have been described as a phenotypically heterogeneous cell type that possess both pro- and anti-tumor properties. Recently, a subset of neutrophils isolated from the peripheral blood mononuclear cell (PBMC) fraction has been described in cancer patients. These low-density neutrophils (LDNs) show a heterogeneous maturation state and have been associated with pro-tumor properties in comparison to mature, high-density neutrophils (HDNs). However, additional studies are necessary to characterize this cell population. Here we show new surface markers that allow us to discriminate between LDNs and HDNs in non-small cell lung cancer (NSCLC) patients and assess their potential as diagnostic/prognostic tool. LDNs were highly enriched in NSCLC patients (median=20.4%, range 0.3-76.1%; n=26) but not in healthy individuals (median=0.3%, range 0.1-3.9%; n=14). Using a high-dimensional human cell surface marker screen, we identified 12 surface markers that were downregulated in LDNs when compared to HDNs, while 41 surface markers were upregulated in the LDN subset. Using flow cytometry, we confirmed overexpression of CD36, CD41, CD61 and CD226 in the LDN fraction. In summary, our data support the notion that LDNs are a unique neutrophil population and provide novel targets to clarify their role in tumor progression and their potential as diagnostic and therapeutic tool.

## Introduction

Lung cancer is the leading cause of cancer deaths worldwide and kills more patients each year than does breast, colon, prostate and pancreatic cancer, combined (1). Most patients are diagnosed at advanced stage with limited treatment options and overall five-year survival rates are below 18% (2). Detection at early stage, when the disease is still localized, significantly increases five-year survival rates to 55%; however, only 16% of lung cancer cases are diagnosed at early stage. For late-stage diagnosis with distant metastases five-year survival rates are as low as 4% (2). The two main lung cancer types are small cell lung cancer (SCLC) and non-small cell lung cancer (NSCLC) (2). NSCLC comprises mainly of lung adenocarcinoma and lung squamous cell carcinoma, representing ~80% of all lung cancer cases (3). Although surgical intervention for early-stage NSCLC can be curative, traditional chemo-and radiotherapy on the one hand, and targeted- and immunotherapy on the other hand, are of limited effectiveness (3). Diagnosis at advanced stage and poor outcome for most patients, highlights the need for novel biomarkers to improve early lung cancer detection and tools to monitor therapy response.

Tumor initiation and progression are commonly accompanied by inflammation (4) and the role of neutrophils in the development of cancer has attracted interest over the last years. Multifaceted and opposing roles of neutrophils in lung cancer have been highlighted (5–7), although the bulk of clinical evidence mostly supports the idea that neutrophils promote cancer progression and have immunosuppressive properties (8). Therefore, our view of neutrophils as a uniform immune population of terminally differentiated cells has given way to the concept that neutrophils are plastic cells that can display distinct morphological, phenotypical and functional properties in health and disease (9, 10). Molecules involved in neutrophil homeostasis are often upregulated in tumors (11–15), leading to neutrophil release from the bone marrow, including immature cells identified by their nuclei shape (9, 13, 16–18). However, the phenotype, relevance and function of immature neutrophils in circulation of tumor patients is not well understood yet. Neutrophil to lymphocyte ratio (NLR) has been shown to correlate with outcome and has been proposed as biomarker for cancer risk assessment and treatment decisions (19, 20). Additional measurement of neutrophil-activating and polarization factors, or identifying neutrophil subpopulations in circulation, released from the bone marrow in response to tumor, would enhance the clinical potential of neutrophils as biomarker for cancer patients.

Low-density neutrophils (LDNs), a neutrophil subtype largely absent in healthy volunteers, have been reported in cancer patients (6, 9, 21, 22) but their function and potential as biomarkers for lung cancer has not been comprehensively evaluated. LDNs consist of mature and immature neutrophil subsets and have been associated with immunosuppressive functions, as opposed to the high-density neutrophils (HDNs) which have anti-tumorigenic properties (9). These findings raise the question which are the distinct features that truly characterize LDNs and separate them from HDNs in the same patient. LDNs have previously also been described as PMN-MDSC or granulocytic-MDSC (myeloid derived suppressor cells) (23), however, additional studies are necessary to define the phenotypic and functional properties of this cell populations in more detail.

In the current study, we focus on the identification of cellular markers that distinguish LDNs from HDNs in NSCLC patients which have the potential to act as novel biomarkers. We show that LDNs are increased in lung cancer patients. Using a high-dimensional flow cytometry screening panel we identified surface markers that are uniquely expressed by the LDN subset in NSCLC patients. Our data support the concept that neutrophils in cancer display diverse phenotypes. Moreover, we provide novel targets to identify and manipulate LDNs and thereby further understand their role in tumor progression and their potential as diagnostic tool.

## Material and Methods

### Study Design

NSCLC patients were recruited from the Department of Internal Medicine, Division of Oncology and Department of Surgery, Division of Thoracic and Hyperbaric Surgery, Medical University of Graz (Graz, Austria) (**Table 1**). Informed consent was obtained from all participants and blood samples were obtained prior to treatment. Healthy volunteers without a history of cancer were recruited as control group. Blood samples were processed within 4 hours after blood draw.

**Table 1:** Participants characteristics.

	All NSCLC
**N**	n = 54
**Age**	66 (45 – 81)
**Sex**	Female n = 21
	Male n = 33
**Smoking**	Yes n = 44
	No n = 9
	Unknown n = 1
**Stages**	I-IIa n = 18
	IIIa n = 8
	Ib n = 6
	IIb n = 6
	IIIb n = 4
	IV n = 0
	Undefined n = 12
**Histology**	Adenocarcinoma n = 31
	Squamous cell carcinoma n = 15
	Undefined NSCLC n = 8
**Therapy**	No prior treatment n = 54
	Treatment n = 0

### Study Approval

The study complied with the Declaration of Helsinki and was approved by the Ethics Committee of the Medical University of Graz (EK-numbers: 30-105 ex17/18, 29-593 ex 16/17 and 17-291 ex 05/06).

### Density Centrifugation

Blood from NSCLC patients and healthy volunteers (10 ml) was collected in EDTA-containing tubes (Greiner). Platelet-rich plasma was removed by centrifugation (300 g, 20 min) and erythrocytes were removed by dextran sedimentation. An equal volume of 3% Dextran T-500 in saline (Sigma Aldrich) was added to the cells, and mixed. Erythrocytes were allowed to sediment for 30 min, and the upper phase containing leukocytes was slowly layered on top of Histopaque 1077 (Sigma Aldrich) in 15 ml tubes. High-density polymorphonuclear leukocytes (PMNL, containing high-density neutrophils (HDNs)) were then separated from low-density peripheral blood mononuclear cells (PBMC; comprising LDNs in cancer patients) by centrifugation (300 g, 20 min, no brake). PBMCs were carefully removed from the interphase to a fresh tube and washed in PBS without Ca^2+^ and Mg^2+^. NH_4_Cl was used to lyse erythrocytes in the high-density fraction (PMNLs). PMNLs were washed twice in PBS without Ca^2+^ and Mg^2+^. PBMCs and PMNLs were resuspended in PBS. Whole blood samples were collected in EDTA-containing tubes, erythrocytes were lysed in NH_4_Cl and cells were washed in PBS without Ca^2+^ and Mg^2+^. Cell viability and cell numbers were determined using an EVE automated cell counter (NanoEntek).

### Cytospins

Cytospins were prepared from the PBMC fractions by centrifugation of 1x10^5^ cells in PBS onto a glass slide (600 g, 5 min) using a Shandon Cytospin 3 and stained immediately using Hemacolor^®^ rapid staining of blood smear according to manufacturer´s instructions (Merck).

### Flow Cytometry

Flow cytometry staining was performed immediately after PMNL and PBMC fractions were obtained or on lysed whole blood samples. In brief, cells were pre-incubated with human TruStain FcX blocking solution (FcBlock, Biolegend) to reduce non-specific binding in staining buffer (SB, PBS + 2% FBS) and incubated at 4°C for 10 min. Subsequently, cells were stained for 20 min at 4°C with CD66b-APC (LDN identification) or CD45-AF700, CD66b-PeCy7, CD36-BV421, CD41-BV785, CD61-FITC, CD226-BV711 and Lox1-PE (marker validation) antibodies in SB. Cells were centrifuged (500 g, 5 min, 4°C), resuspended in 200 µL of SB and washed again with SB. Propidium iodide (PI, 5 min, RT) or fixable viability dye eFluor™ 780 (FVD, eBioscience) (30 min, 4°C) was used to stain dead cells. FVD stained cells were fixed with IC fixation buffer (eBioscience) (10 min, 4°C), centrifuged and resuspended in 100 µL SB. Samples were measured on a FACS Canto II or BD LSR II Fortessa (BD Biosciences).

### Flow Cytometry – LEGENDScreen Neutrophil Surface Marker Screening

PBMC and PMNL fractions were isolated by density centrifugation and stained for flow cytometry analysis. Briefly, cells were incubated with FVD eFluor™ 780 (eBioscience) (30 min, 4°C), washed twice with SB and pre-incubated with FcBlock (Biolegend) (10 min, 4°C). Subsequently, cells were stained with the following antibody master mix (antibody and clone details, see **Supplementary Table 1**): PBMC panel; CD45-AF700, CD3-PECy5, CD4-BUV395, CD8-BUV496, CD19-FITC, CD14-BUV605, CD66b-APC, Siglec8-PECy7. PMNL panel; CD45-AF700, CD66b-APC, Siglec8-PeCy7 (30 min, 4°C). Cells were then distributed (3x10^5^ cells/well) and stained according to the LEGENDScreen™ Human PE Kit (Biolegend) protocol. PBMCs and PMNLs from 6 NSCLC patients were isolated to acquire enough cells for all 361 markers (including 10 isotype controls). Cells were measured on a BD LSR II Fortessa (BD Biosciences). 33 markers were excluded from analysis due to low live neutrophil counts (<100 cells) or abnormal FSC/SSC properties. 328 samples were included in the final analysis based on their quality after data acquisition.

### InfinityFlow Analysis

The R Package InfinityFlow without background correction was used for the prediction of marker co-expression and the two-dimensional projection of the data using Uniform Manifold Approximation and Projection (UMAP) plots (standard parameters as stated in the package description) (24). 328 fcs files of the LEGENDScreen were used as input. Graphs were drawn with the Packages ggplot2_3.3.3, pheatmap_1.0.12 and corrplot_0.84 (R-Version 4.0.3).

### Statistical Analysis

Flow cytometry data were reported as % of cells, % of CD45^+^ cells or % of CD66b^+^ cells and as Geometric Mean (GeoMean) and Median Fluorescence Intensity (MFI). Compensation of flow cytometry data was performed using single stains. Cut-offs for background fluorescence were based on the ‘fluorescence minus one’ (FMO) strategy (25). Data were analyzed using FlowJo software (TreeStar).

Statistical analyses were performed using GraphPad Prism 6.1 (GraphPad Software). All data were tested for Gaussian distribution of variables using the Shapiro-Wilk normality test. Significant differences between healthy controls and patient samples with normal distribution were determined using unpaired student’s t-tests with Welch´s correction, otherwise Mann-Whitney test was applied. For comparison between samples from the same patient, paired t-test (parametric) or Wilcoxon matched-pairs test (non-parametric) was applied. Results are expressed as the mean ± S.E.M. A p-value <0.05 was considered statistically significant.

## Results

### Low-Density Neutrophils Are Expanded in Patients With NSCLC

The presence of granulocytes in the mononuclear fraction of peripheral blood has previously been reported in the blood of patients with cancer including renal carcinoma, head and neck cancer, pancreatic cancer, colon cancer and breast adenocarcinoma (26–28). More recently, LDNs have also been reported in lung cancer patients (6, 21, 22, 29).

To determine whether LDNs were also present in this NSCLC patient cohort, blood samples were collected from NSCLC patients, regardless of disease stage. PBMCs were isolated from peripheral blood of NSCLC patients and healthy controls by density gradient centrifugation. As controls, PBMCs and PMNLs from healthy donors were used.

**Figure 1:**
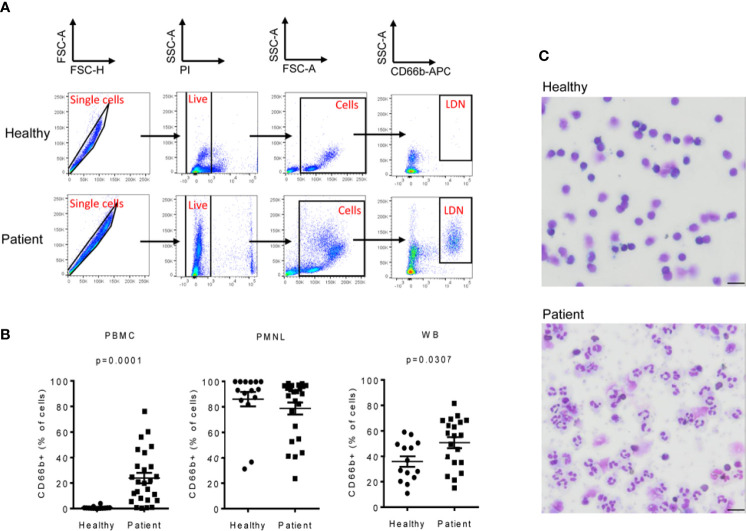
Low-density neutrophils (LDNs) are elevated in patients with NSCLC. PBMC and PMNL fractions were isolated from peripheral blood of patients with NSCLC by density gradient centrifugation. Immediately after isolation, PBMCs, PMNLs and whole blood cells were stained with CD66b antibody to identify LDNs. **(A)** Representative polychromatic dot plots demonstrating the gating strategy employed to identify LDN cell content in the PBMC fraction of peripheral blood of healthy controls and NSCLC patients. Initial gate is to eliminate doublets from the analysis followed by gating on live (PI-). Total cells were gated based on their forward and side scatter properties and LDNs were identified as CD66b^+^ cells. **(B)** LDNs were quantified as the percentage of CD66b^+^ cells in the PBMC fraction of peripheral blood of 26 NSCLC patients and 14 healthy controls. Each symbol represents an individual donor. The percentage of CD66b^+^ cells in the PMNL fraction and whole blood (WB) was also evaluated. **(C)** Representative cytospin images of the PMBC fractions of a healthy donor showing no LDNs and a NSCLC patient with heterogeneous LDNs including mature (segmented nucleus) and immature neutrophils (band cells). Pictures were acquired with a 40X objective, bar length 20µM. Statistical differences were assessed by using unpaired student´s t-test with Welch´s correction or Mann-Whitney test and data are expressed as the mean ± S.E.M.

LDNs were identified as CD66b^+^ cells in the PBMC fraction of NSCLC patients (**Figures 1A–C** and **Supplementary Figures 1A, B**). We found that the LDN fraction was significantly increased in NSCLC patients (median=20.4%, range 0.3-76.1%; n=26) when compared to healthy volunteers. LDNs were almost absent in healthy controls (median=0.3%, range 0.1-3.9%; n=14) (**Figure 1B**). No differences were found in the percentage of CD66b^+^ cells in the PMNL fraction of NSCLC patients and healthy donors (**Figure 1B**), however, the percentage of CD66b^+^ cells was significantly increased in whole blood of patients (median=56.4%, range 15.1-81.5%) vs. controls (median=32.9%, range 10.9-59.1%) (**Figure 1B**) which might reflect the higher number of LDNs in patients.

In addition, morphological analyses showed that, LDNs were absent in the PBMC fraction of healthy volunteers, whereas in NSCLC patients, LDNs were heterogeneous and contained segmented as well as morphologically immature (banded and ring-shaped) neutrophils (**Figure 1C**) as has previously been reported (9).

### Unbiased Surface Marker Screen to Identify LDN Specific Markers Using Flow Cytometry

We next aimed to characterize the phenotype of LDNs in NSCLC patients with the intention to find specific surface markers that discriminate LDNs and HDNs. Previous studies have mostly separated LDNs from HDNs based on the expression of known myeloid cell and neutrophil maturation markers (30–33). Our study aimed to analyze a wider range of surface markers, most of which have not been previously reported to be expressed by neutrophils.

**Figure 2:**
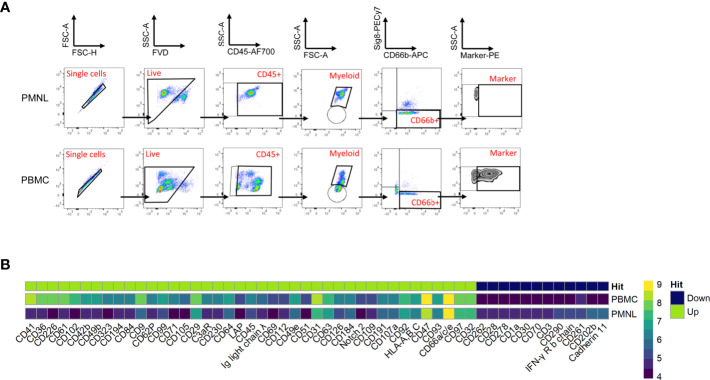
Comprehensive surface marker screening in PMNLs and PBMCs from NSCLC patients. PBMCs and PMNLs were isolated from peripheral blood of NSCLC patients by density gradient centrifugation. Cells were initially stained with a backbone panel including antibodies against CD45, CD66b and Siglec8 and then distributed and stained with 361 anti-human PE-conjugated variable antibodies (Legend Screen, Biolegend). **(A)** Representative flow cytometry dot plots demonstrating the gating strategy employed to identify the PE-conjugated cell surface markers in the HDN and LDN fractions. Initial gate is to eliminate doublets from the analysis followed by gating on live (FVD) and CD45^+^ cells. The myeloid population (containing neutrophils and eosinophils) was gated based on its forward and side scatter properties. Neutrophils (HDNs in PMNL fraction and LDNs in PBMC fraction) were identified as CD66b^+^ Siglec8^-^ cells and PE channel was used to analyze the individual surface markers. **(B)** Expression heatmap of marker hits. Hits were defined by fold change marker expression (GeoMean) with fold change > 2 and < 0.5 (ratio LDN/HDN) (see details **Supplementary Table 2**). Heatmap scale represents the GeoMean and ‘hits’ show the differentially regulated markers in the LDNs (PBMC gated) vs HDNs (PMNL gated); green-overexpressed, dark blue-downregulated.

**Figure 3:**
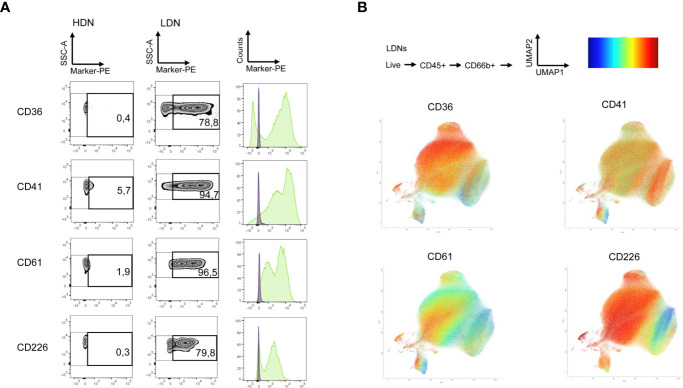
LDNs show heterogenous expression of top screening hits. **(A)** Selected surface antigens with the most pronounced differences in expression between HDNs and LDNs. Zebra plots representing CD36, CD41, CD61 and CD226, respectively, in the HDN and LDN fractions are presented. Histograms showing the fluorescence intensity in the HDNs (violet) and LDNs (green) are also displayed. **(B)** The results of the LegendScreen were used as an input for the InfinityFlow pipeline. UMAP plots demonstrating heterogeneity of CD36, CD41, CD61 and CD226 expression in LDNs were created based on the predictions of the pipeline expression level high (red), low (blue).

For this purpose, we used the human LEGENDScreen (Biolegend) and measured 328 surface markers in combination with ‘backbone’ markers that define neutrophils and performed InfinityFlow analysis (24). Briefly, the expression of PE-conjugated markers was used to predict the specific expression of each exploratory marker on all single cell events acquired through the LEGENDScreen *via* non-linear regression using the expression of the backbone markers. PBMCs and PMNLs were isolated from peripheral blood of NSCLC patients and live, CD66b^+^ Siglec8^-^ cells were identified as LDNs and HDNs in the PBMC and PMNL fractions, respectively (**Figure 2A**, Siglec8 was used to separate neutrophils (Siglec8^-^) and eosinophils (Siglec8^+^) within the CD66b^+^ gate). 53 markers were up or downregulated (defined by LDN/HDN fold change in GeoMean - lower than 0.5 or higher than 2) in LDNs when compared to HDNs (**Figure 2B** and **Supplementary Table 2**) and the top four upregulated hits (CD36 fold change=31.1, CD41 fold change=6.7, CD61 fold change=20.1, CD226 fold change=27.8) showed a fold change above 5 (**Figures 3A, B** and **Supplementary Table 2**). UMAP plots of the LDN population confirmed the observations made by manual gating and moreover, revealed a heterogeneous expression of CD36, CD41, CD61 and CD226 (**Figure 3B**).

### LDNs of NSCLC Patients Reveal Distinct Surface Marker Expression Profile

**Figure 4:**
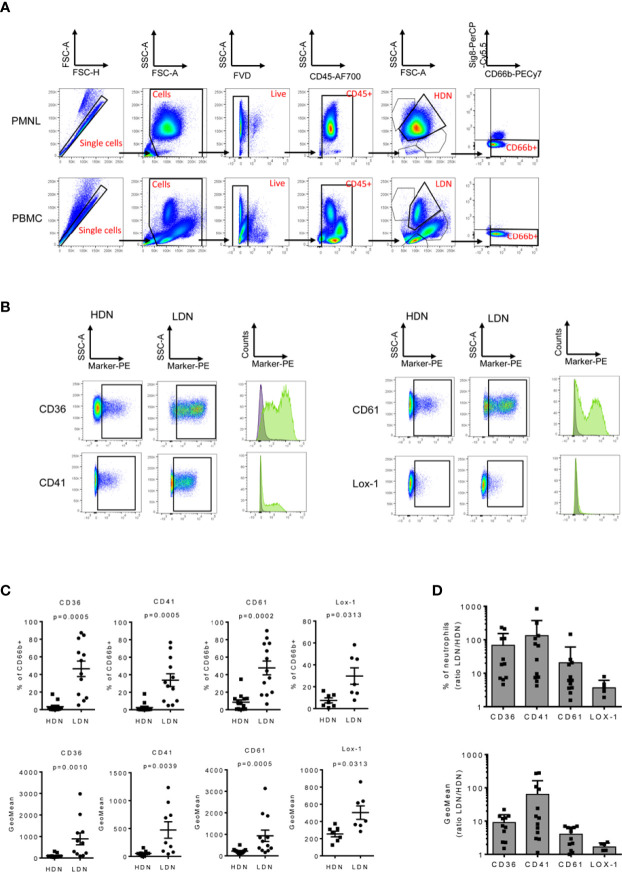
LDNs overexpress CD36, CD41, CD61 and Lox1. PMBCs and PMNLs were isolated from 13 NSCLC patients and stained to validate the surface marker expression by flow cytometry. **(A)** Representative flow cytometry dot plots demonstrating the gating strategy. Initial gate is to eliminate doublets from the analysis followed by gating on total cells, live (FVD) and CD45^+^ cells. HDNs and LDNs were gated base on its forward and side scatter properties. Neutrophils were identified as CD66b^+^ Siglec8^-^ cells **(B)** Representative dot plots and histograms showing an increased expression of CD36^+^, CD41^+^, CD61^+^ and Lox1^+^ in the LDN fraction of cancer patients. **(C)** Quantitative analysis of the marker expression as % of CD66b^+^ cells and GeoMean in the HDN and LDN subsets in NSCLC patients. **(D)** The LDN/HDN ratio of the different markers was also analyzed. Statistical differences were assessed by using paired t-tests or Wilcoxon matched-pairs and data are expressed as the mean ± S.E.M.

To validate the surface marker screening results, some of the highest expressed markers in the LDN fraction were selected, including CD36, CD41, CD61 and CD226 (**Figure 3A**) respectively. Unbiased InfinityFlow protein expression analysis revealed heterogenous expression patterns of all four markers within the LDN population (**Figure 3B**). Lox-1 was also included in the validation panel since it has been suggested to be a distinctive marker of the LDN population in cancer patients (34), although no difference in expression was observed between HDNs and LDNs in the surface marker screen (fold change=1.1, **Supplementary Figure 2**). Flow cytometry was used to evaluate the expression of the selected markers in the PBMC and PMNL fractions in a cohort of 13 NSCLC patients (**Figures 4A–D**). We confirmed that LDNs (defined as CD66^+^ Siglec8^-^ cells) showed a significantly higher proportion of CD36 (median=48.2%, range 5.1-87.4%), CD41 (median=29.2%, range 5.3-70.8%), CD61 (median=44.4%, range 16.8-90.2%) and Lox1 (median=22.1%, range 7.7-58.3%) when compared to HDNs (**Figures 4C, D**). These results were also reflected as an increase in the GeoMean of the selected markers in LDNs vs. HDNs (**Figure 4C**). CD226 (median=4.6%, range 0.6-35.2%, n=6) also showed increased expression on LDNs vs. HDNs, however this marker was excluded due to weak separation of negative and positive populations (**Supplementary Figure 3**). Further, we observed an increased LDN/HDN ratio of all respective markers expressed as % of CD66b^+^ cells and GeoMean (**Figure 4D**). LDNs have been described as a population consisting of mature and immature neutrophil subsets. In order to address the maturation status of LDNs in our study cohort, we analyzed the expression of some myeloid and maturation markers including CD10, CD16, CD15 and CD11b in the LDN and HDN fractions of 7 NSCLC patients (**Supplementary Figure 4A**). We did not find significant differences in the expression of any of the markers when comparing HDNs vs LDNs (**Supplementary Figure 4A**). The analysis of the expression of CD10 in the LDNs and HDNs of 7 NSCLC patients, revealed that the vast majority of the HDN fraction expressed CD10, confirming the nature of mature neutrophils in this fraction (**Supplementary Figure 4B**). In contrast, LDNs presented more variability in the proportion of CD10^-^ vs CD10^+^ between different patients (**Supplementary Figure 4B**). Further studies analyzing the co-expression of neutrophil maturation markers and the surface markers CD36, CD41 and CD61 in larger patient cohorts are necessary. Taken together, these data suggest that expression levels of the surface markers CD36, CD41, CD61 and Lox-1 are significantly higher on LDNs when compared to HDNs.

### LDN Markers Are Co-Expressed in LDNs of NSCLS Patients

**Figure 5:**
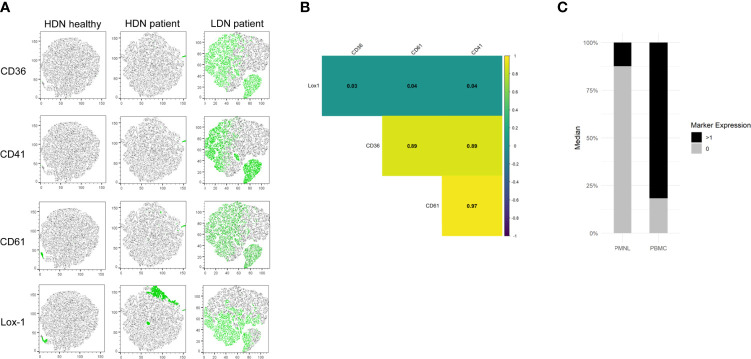
CD36, CD41, CD61 and Lox-1 are co-expressed in LDNs from NSCLC patients. **(A)** Representative tSNE visualization of HDNs and LDNs demonstrates almost absent expression of CD36, CD41, CD61 and Lox-1 in HDNs from healthy donors and NSCLC patients while a heterogeneous expression of the markers was detected in LDNs from NSCLC patients. **(B)** Correlation analysis of the expression of the four studied markers indicating no correlation of Lox-1 with the rest of the markers and high correlation between CD36, CD41 and CD61 in one representative patient. (all results statistically significant/p<0.01). **(C)** Proportions of positive marker expression (black) and negative marker expression (grey) in the PMNL and PBMC fractions of NSCLC patient.

In order to investigate whether the new identified LDN markers are co-expressed, we used t-distributed stochastic neighbor embedding (tSNE) plots and correlation analysis. tSNE plots were generated from the HDN and LDN populations of a representative healthy and NSCLC patient. We observed that none of the investigated markers was expressed in the HDNs of the healthy donor (**Figure 5A**). Moreover, when we compared HDNs and LDNs of a NSCLC patient we observed a heterogeneous expression of the markers in the LDN population, with some cells sharing expression of all the markers, other cells being positive for some of the markers and a subset of cells showing no positive signal for any of the antigens. No positive signal of CD36, CD41 and CD61 was observed in the HDNs (**Figure 5A**), however, it is important to note that Lox-1 showed expression in a subset of cells in the HDNs of the patient (**Figure 5A**).

Further analysis revealed a strong correlation between CD36, CD41 and CD61 expression while Lox-1 did not show correlation with any of the other markers (**Figure 5B**). Moreover, we observed that using the selected markers we can capture around 80% of all LDNs (**Figure 5C**).

## Discussion

The presence of myeloid cells in the complex scenery of cancer has shown to have pivotal roles. In this context, neutrophils have recently attracted attention in terms of diversity and functionality. It is getting evident that neutrophils are heterogeneous and consist of diverse, dynamic subpopulations that have distinct phenotypes and can oppose or enhance cancer progression. It is possible that the ratio between the different neutrophil subpopulations, and the nature of their activity can define their overall role in cancer.

Here we report that LDNs are increased in NSCLC patients when compared to healthy individuals independent of stage. It has to be noted that within the sampled NSCLC patients, LDN presence ranges from 0.3-76.1% of all cells in the PBMC fraction. This indicates that LDN abundance is heterogenous and might correlate with tumor stage as previously reported (22, 29). The clinical significance of the presence of LDNs in the circulation of cancer patients is still unknown. Sagiv and colleagues have proposed that circulating neutrophils, besides their different density properties, might possess functional differences being the ‘normal’ HDNs anti-tumorigenic, whereas LDNs are associated with pro-tumor activity (9).

Current limitations to further exploit the power of LDNs as diagnostic and prognostic factor is the relatively high work load to isolate them (sucrose gradient centrifugation followed by flow cytometry staining) and the necessity to process the blood samples within a few hours after blood draw; main limitations to perform these experiments in a clinical setting. On this regard, some efforts have been made to further characterize different neutrophil subpopulations in circulation.

Most studies have been focused on myeloid and maturation markers (such as CD10, CD11b, CD15 and CD16, CXCR4, PDL-1) (22, 33, 35) and there is a lack of unbiased analysis of neutrophil heterogeneity in circulation in the setting of cancer. This is most likely because unbiased gene expression analysis is difficult to perform on neutrophils due to low RNA abundance in mature cells. One exception is the study by Condamine et al. identifying Lox1 as a LDN surface marker using a gene expression array (34). Although the immature status of neutrophils was initially thought to be directly related with their immunosuppressive and tumor-supportive role, in contrast with the cytotoxic function of mature neutrophils, multiple evidences collected in both human and animal models show that mature neutrophils can display immunosuppressive functions as well (9, 10).

We performed an unbiased high-dimensional flow cytometry surface marker screen on CD66b^+^Siglec8^-^ cells isolated from the PBMC (LDNs) and PMNL (HDNs) fractions of NSCLC patients with the aim to identify markers which distinguish LDNs and HDNs. Although the power of our screen analysis is limited due to sample size, UMAP and correlation analysis together with flow cytometry-based validation of the top hits, revealed new cellular markers that are significantly overexpressed in LDNs vs. HDNs. The top hits from the screen (CD36, CD41, CD61 and CD226) could be confirmed to be overexpressed in the LDN fraction of NSCLC patients when compared to HDNs.

CD36 is a widely studied transmembrane glycoprotein that functions as a scavenger receptor (36). It is involved in multiple cellular functions including lipid uptake, immunological recognition, inflammation, molecular adhesion, and apoptosis (36). CD36 is expressed on the cell surface of multiple cell types, including macrophages, monocytes, dendric cells, microvascular endothelial cells, retinal epithelial cells, adipocytes, platelets, enterocytes, microglial cells and podocytes (36, 37). CD36 expression has been also reported in tumor cells, stromal and immune cells in tumor tissues (37). In cancer, CD36 plays important roles in lipid homeostasis, immune response, angiogenesis, adhesion and metastasis (37). CD41, also known as αIIb integrin, is expressed on platelets and megakaryocytes (38, 39). Its expression has also been reported in hematopoietic progenitors in the embryo, fetus and adult of various species and during early stages of hematopoietic differentiation (40). CD61 (integrin β3) is expressed on megakaryocytes, besides its expression on human plasmacytoid dendritic cells. It is involved in the uptake of apoptotic cells and induction of immune tolerance (41, 42). CD41 and CD61 are often associated in a complex to form the integrin GPIIb/IIIa, which plays a major role in platelet function, acting as a receptor for several adhesion molecules, including fibronectin, fibrinogen, vitronectin and Willebrand factor (38, 40). The CD41/CD61 complex is required for platelet aggregation and clotting (38).

These novel LDN markers clearly describe this neutrophil subset in circulation of NSCLC patients. LDN presence has also been reported in the PBMCs of human patients suffering from other pathologies including autoimmune disorders and asthma, systemic and local infection, HIV, dermatomyelosis and malaria (43–53). Although the first evidence suggested that LDNs were a neutrophil subset present only under pathological conditions, LDNs have also been identified during pregnancy and in newborns (30, 54, 55). CD36, CD41 and CD61 has expression to be evaluated in LDNs in these pathologies and during pregnancy to further understand LDN heterogeneity and function. Further studies using larger cohorts and including patients in different stages of disease and under treatment will be necessary to confirm the potential use of these identified markers as prognostic biomarkers or to monitor treatment. Moreover, the development of whole blood-based protocols for the identification of the suggested markers is a crucial step towards future applications of the use of these markers as diagnostic and prognostic tools.

It has to be noted that, based on our data, low marker expression on HDNs was observed in some patients. This further supports the concept of neutrophil plasticity and the idea that HDNs and LDNs represent a continuum in the spectrum of phenotypes neutrophils can acquire. In this context, a recent report showed than HDNs from cancer patients can be retrieved in the LDN fraction and a fraction of the LDNs can be retrieved in the HDN fraction (22). This is in accordance with a previous study that described that in tumor-bearing mice HDNs can become LDNs and LDNs can switch toward HDNs, *in vivo* and *ex vivo* (9). Further studies including the new suggested LDN markers CD36, CD41 and CD61 in addition to neutrophil maturation markers would help to improve our knowledge regarding the origin and function of LDNs.

## Data Availability Statement

The original contributions presented in the study are included in the article/**Supplementary Material**. Further inquiries can be directed to the corresponding author.

## Ethics Statement

The studies involving human participants were reviewed and approved by Ethics Committee of the Medical University of Graz. The patients/participants provided their written informed consent to participate in this study.

## Author Contributions 

PV-C, JK and KM designed and supervised the research. PV-C and KM performed the experiments and analyzed the results. OK performed bioinformatic analysis. SR, MK, AS and ZNM assisted with the performance of the experiments. LB, JL, MF and MP provided the clinical samples and maintained the clinical database. PV-C and OK performed statistical analyses. PV-C and OK crafted the figures. PV-C, KM, OK, RS, AMH, AH and JK interpreted the data and provided technical support. PV-C and JK wrote the manuscript. All authors critically revised and commented on the manuscript. All authors contributed to the article and approved the submitted version.

## Funding

This work was supported by the OENB Anniversary Fund (17584) and FFG-Bridge 1 grant (871284). PhD candidates PV-C, OK, SR, MK, AS, and ZNM received funding from the FWF [doctoral programs: DK-MOLIN (W1241) and DP-iDP (DOC-31)] or BioTechMed and were trained within the frame of the PhD Program Molecular Medicine of the Medical University of Graz. Work in the lab of RS is funded by FWF grants P33325 and KLI887.

## Conflict of Interest

The authors declare that the research was conducted in the absence of any commercial or financial relationships that could be construed as a potential conflict of interest.

## Publisher’s Note

All claims expressed in this article are solely those of the authors and do not necessarily represent those of their affiliated organizations, or those of the publisher, the editors and the reviewers. Any product that may be evaluated in this article, or claim that may be made by its manufacturer, is not guaranteed or endorsed by the publisher.
