# Supplementary material for: Identification of Novel Low-Density Neutrophil Markers Through Unbiased High-Dimensional Flow Cytometry Screening in Non-Small Cell Lung Cancer Patients
Source: Front Immunol. 2021 Aug 13;12:703846. doi: 10.3389/fimmu.2021.703846 (PMC8414579; doi:10.3389/fimmu.2021.703846)
Supplement: Supplementary file 1 [file DataSheet_1.docx]

**
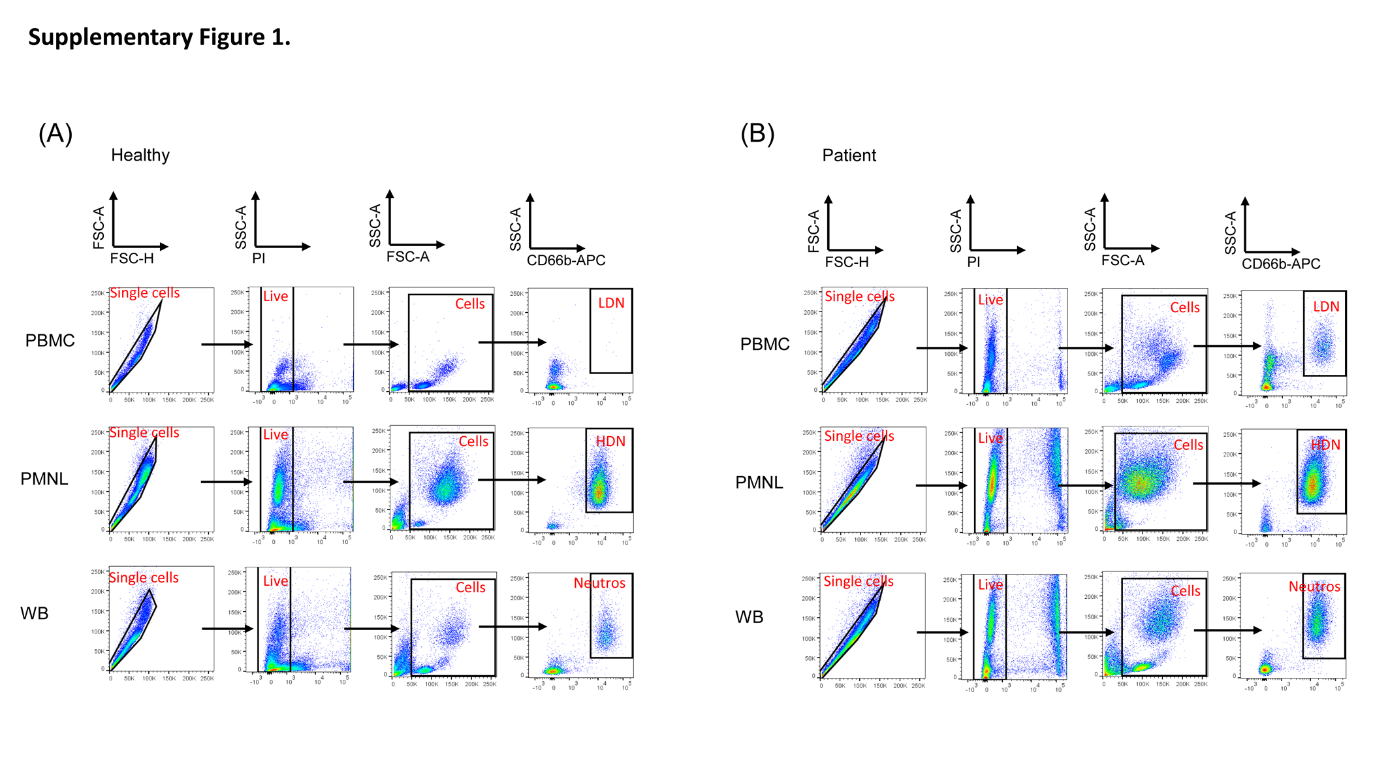
SUPPL. FIG 1** | **Flow cytometry gating strategies for PBMCs, PMNLs and whole blood.** Schematic presentation of the gating strategy for analysis of LDNs, HDNs and total neutrophils in PBMC fraction, PMNL fraction and whole blood (WB) of healthy volunteers **(A)** and NSCLC patients **(B)**. Cells were pre-gated for single cells and dead cells were excluded using propidium iodide (PI). Total cells were selected based on their forward and side scatter properties and LDNs, HDNs and total neutrophils were determined as CD66b^+^.


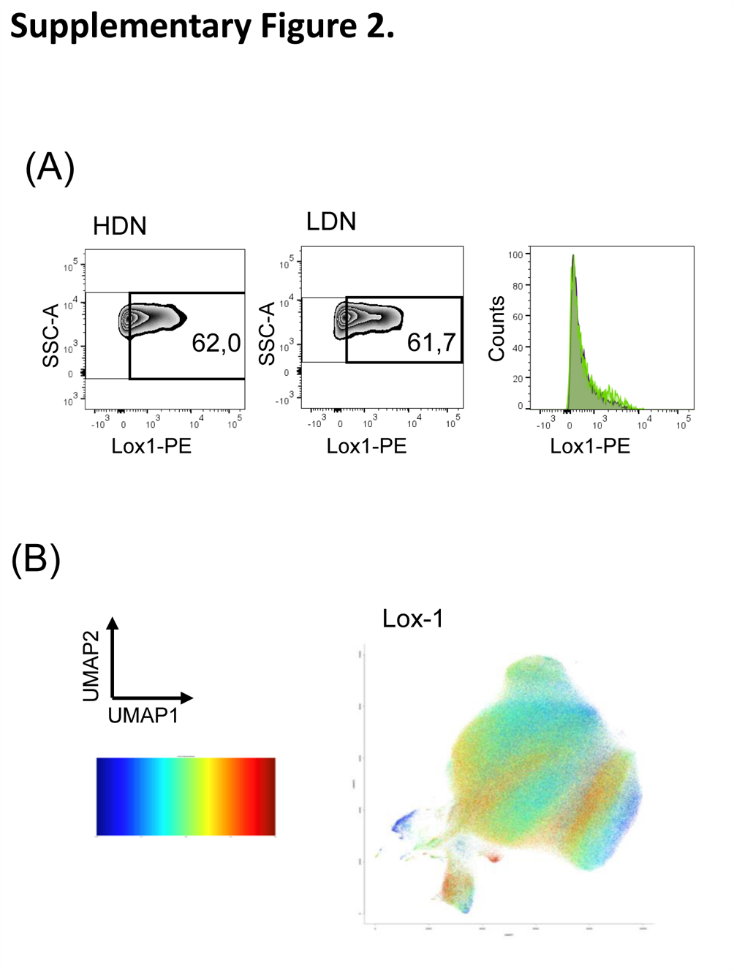


**SUPPL. FIG 2** | **Surface marker screen showed no difference in Lox-1 expression between HDNs and LDNs.** **(A)** Zebra plots and histograms representing Lox-1 expression in HDNs (dark green) and LDNs (light green) are displayed. **(B)** UMAP dimensionality reduction demonstrates heterogenous Lox-1 expression within the LDN fraction based on the surface marker screen data.


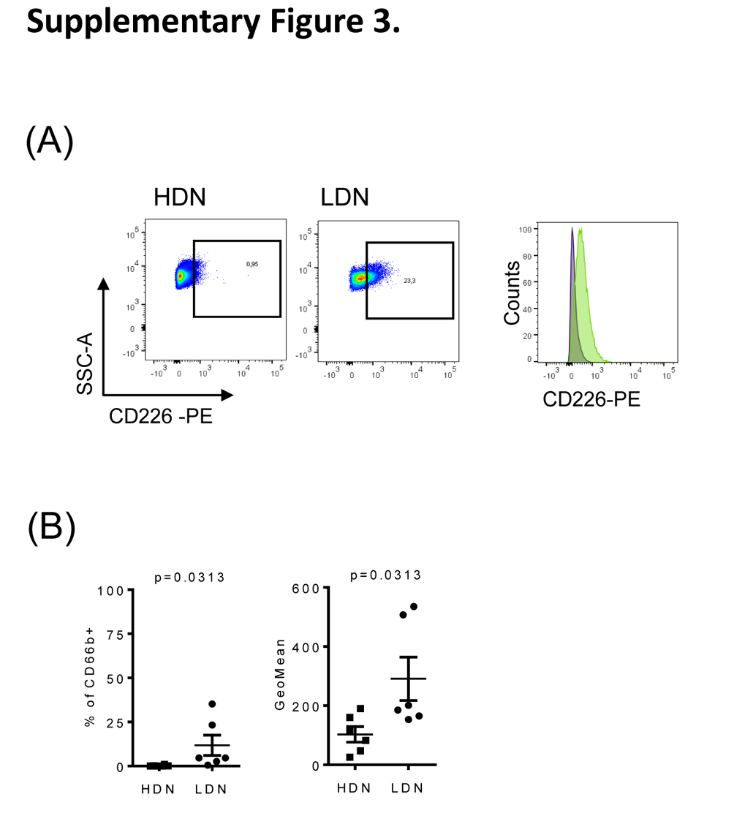


**SUPPL. FIG 3** | **LDNs overexpress CD226.** **(A)** Representative plots and histograms showing increased expression of CD226 in the LDN (light green) fraction of NSCLC cancer patients. **(B)** Quantitative analysis of the markers expression as % of CD66b^+^ cells and GeoMean in the HDN and LDN subsets in NSCLC patients. Statistical differences were assessed using Wilcoxon matched-pairs and data are expressed as the mean ± S.E.M, n=6.


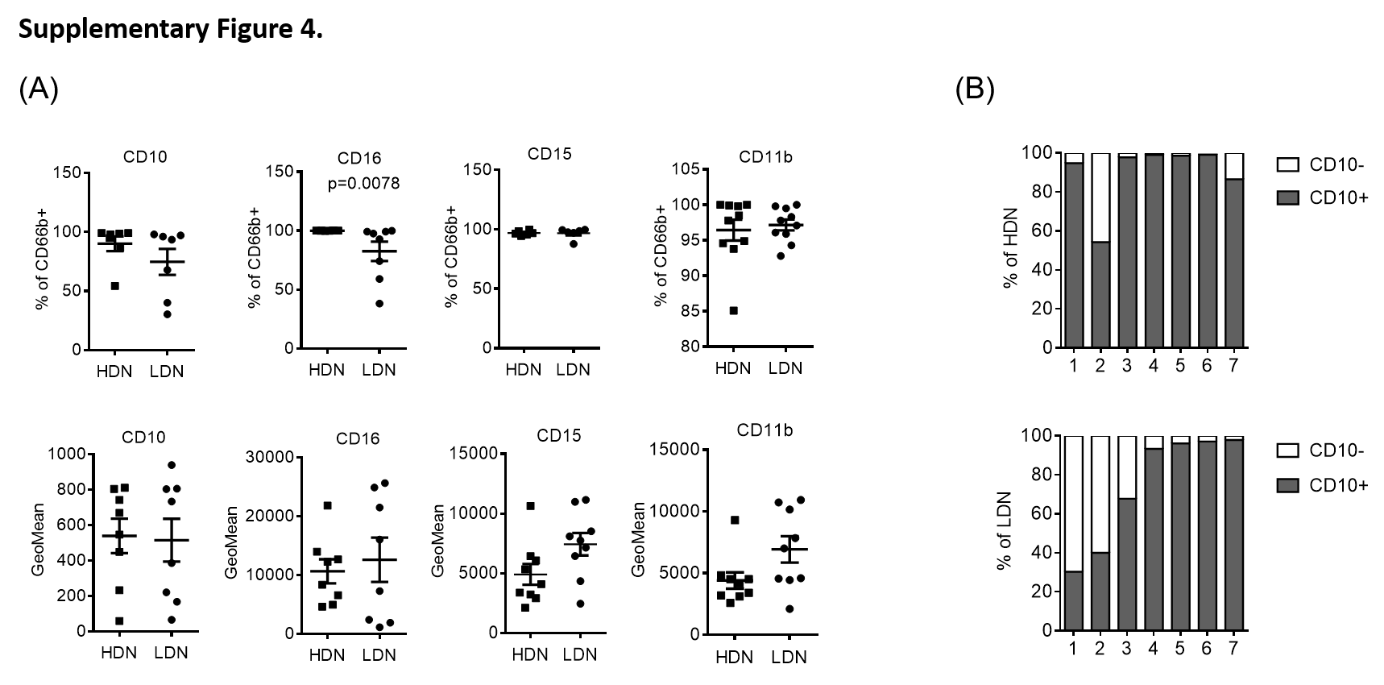


**SUPPL. FIG 4** | **Neutrophil maturation markers in LDNs and HDNs.** **(A)** Quantitative analysis of the maturation markers (CD10, CD16, CD15 and CD11b) expression as % of CD66b^+^ cells and GeoMean in the HDN and LDN subsets in NSCLC patients. **(B)** Proportions of CD10^+^ and CD10^-^ cells out of the HDNs and LDNs of 7 individual NSCLC patients. Statistical differences were assessed by paired t-tests or using Wilcoxon matched-pairs and data are expressed as the mean ± S.E.M

**Supplementary Table 1. List of flow cytometry antibodies used in this study**

| Antigen | Fluorophore | Clone | Company | Catalog # | Panel |
| --- | --- | --- | --- | --- | --- |
| CD45 | AF700 | HI30 | Biolegend | 304024 | Screening, validation |
| CD3 | PE-Cy5 | UCHT1 | Biolegend | 300410 | Screening |
| CD3 | BV510 | UCHT1 | Biolegend | 300447 | Validation |
| CD4 | BUV395 | SK3 | Biolegend | 563550 | Screening |
| CD8 | BUV496 | RPA-T8 | Biolegend | 564804 | Screening |
| CD66b | APC | G10F5 | Biolegend | 305118 | LDN staining, screening, validation |
| CD66b | PE-Cy7 | G10F5 | Biolegend | 305116 | Validation |
| Siglec-8 | PE-Cy7 | 7C9 | Biolegend | 347112 | Screening, validation |
| Siglec-8 | PerCP-Cy5.5 | 7C9 | Biolegend | 347108 | Validation |
| CD19 | FITC | HIB19 | Biolegend | 302206 | Screening |
| CD14 | BUV605 | M5E2 | Biolegend | 301834 | Screening |
| CD61 | FITC | VI-PL2 | Biolegend | 336403 | Validation |
| CD226 | BV711 | 11A8 | Biolegend | 338333 | Validation |
| CD36 | BV421 | 5-271 | Biolegend | 336229 | Validation |
| CD41 | BV785 | HIP8 | Biolegend | 303743 | Validation |
| Lox-1 | PE | 15C4 | Biolegend | 358603 | Validation |
| LEGEND Screen | PE | - | Biolegend | 700007 | Screen |

**Supplementary Table 2. GeoMean, MFI and percentage of expression (data expressed as % of CD66b+, % of CD45+ and % of live) of hits identified in screen. Hits were defined by fold change marker expression (GeoMean) with fold change > 2 and < 0.5 (ratio LDN/HDN)**

| Marker | PBMC | | | | | PMNL | | | | |
| --- | --- | --- | --- | --- | --- | --- | --- | --- | --- | --- |
|  | Geo Mean | MFI | Fq of CD66b | Fq of CD45 | Fq of live | Geo Mean | MFI | Fq of CD66b | Fq of CD45 | Fq of live |
| CD41 | 4992 | 6836 | 94.7 % | 56.4 % | 55.8 % | 136 | 102 | 5.69 % | 2.89 % | 2.80 % |
| CD36 | 2679 | 4407 | 78.8 % | 25.6 % | 24.7 % | 86,1 | 82,2 | 0.42 % | 0.40 % | 0.40 % |
| CD226 | 2123 | 2095 | 98.1 % | 8.95 % | 6.98 % | 76,5 | 71,9 | 0.28 % | 0.24 % | 0.23 % |
| CD61 | 2427 | 2854 | 96.5 % | 61.4 % | 60.9 % | 121 | 104 | 1.97 % | 1.11 % | 1.07 % |
| CD102 | 1192 | 1275 | 71.2 % | 17.6 % | 16.9 % | 79,9 | 75,8 | 0.23 % | 0.22 % | 0.22 % |
| CD42b | 904 | 728 | 59.2 % | 35.6 % | 35.2 % | 94,3 | 89,9 | 0.36 % | 0.19 % | 0.18 % |
| CD49b | 741 | 622 | 67.0 % | 41.9 % | 41.6 % | 106 | 95,1 | 1.61 % | 0.83 % | 0.81 % |
| CD323 | 521 | 429 | 61.6 % | 16.3 % | 15.7 % | 75,8 | 73,2 | 0.13 % | 0.12 % | 0.12 % |
| CD194 | 587 | 437 | 69.2 % | 6.24 % | 6.77 % | 92,9 | 62,9 | 3.05 % | 2.71 % | 2.69 % |
| CD84 | 680 | 535 | 63.0 % | 16.7 % | 16.1 % | 110 | 105 | 1.90 % | 1.81 % | 1.81 % |
| CD9 | 1980 | 3406 | 70.6 % | 7.83 % | 7.66 % | 357 | 246 | 32.4 % | 20.2 % | 19.3 % |
| CD62P | 660 | 488 | 69.6 % | 41.8 % | 41.3 % | 122 | 111 | 1.77 % | 0.95 % | 0.92 % |
| CD99 | 873 | 760 | 86.6 % | 9.42 % | 9.18 % | 164 | 133 | 11.1 % | 6.17 % | 5.95 % |
| CD71 | 404 | 183 | 34.8 % | 9.23 % | 8.85 % | 81,4 | 79,6 | 0.36 % | 0.35 % | 0.35 % |
| CD105 | 391 | 278 | 55.2 % | 1.19 % | 1.19 % | 80,9 | 73,2 | 0.32 % | 0.31 % | 0.30 % |
| CD29 | 3322 | 3497 | 100.0 % | 62.0 % | 61.5 % | 850 | 752 | 83.3 % | 0.44 % | 0.15 % |
| C3aR | 458 | 409 | 68.8 % | 6.90 % | 8.92 % | 129 | 96,4 | 8.13 % | 7.23 % | 7.14 % |
| CD230 | 452 | 351 | 58.4 % | 15.5 % | 15.0 % | 135 | 117 | 8.44 % | 8.02 % | 8.02 % |
| CD64 | 792 | 424 | 60.4 % | 6.27 % | 6.07 % | 247 | 203 | 21.4 % | 11.2 % | 10.7 % |
| LAP | 214 | 139 | 17.3 % | 1.92 % | 1.85 % | 69,9 | 61,6 | 0 % | 0 % | 0 % |
| CD45 | 664 | 593 | 98.5 % | 60.2 % | 59.2 % | 222 | 188 | 13.5 % | 7.14 % | 6.87 % |
| Ig light chain λ | 339 | 249 | 35.6 % | 3.60 % | 9.25 % | 121 | 107 | 2.17 % | 1.89 % | 1.83 % |
| CD69 | 216 | 161 | 18.0 % | 1.75 % | 1.70 % | 77,5 | 69,4 | 0 % | 0 % | 0 % |
| CD112 | 234 | 151 | 28.3 % | 2.67 % | 2.67 % | 93 | 79,6 | 2.29 % | 2.12 % | 2.06 % |
| CD49e | 780 | 650 | 92.3 % | 8.92 % | 7.28 % | 354 | 326 | 59.5 % | 53.3 % | 52.9 % |
| CD51 | 162 | 122 | 12.6 % | 3.10 % | 2.99 % | 77,8 | 75,8 | 0.27 % | 0.25 % | 0.25 % |
| CD31 | 3598 | 3469 | 100.0 % | 61.0 % | 60.4 % | 1742 | 1788 | 99.9 % | 51.9 % | 50.9 % |
| CD63 | 1530 | 1384 | 99.9 % | 62.2 % | 61.7 % | 745 | 561 | 88.9 % | 49.4 % | 47.9 % |
| CD126 | 421 | 420 | 85.0 % | 8.40 % | 8.40 % | 209 | 199 | 9.03 % | 8.45 % | 8.28 % |
| CD184 | 524 | 465 | 81.8 % | 19.9 % | 19.2 % | 261 | 225 | 45.5 % | 43.3 % | 43.3 % |
| Notch 2 | 150 | 143 | 3.10 % | 0.32 % | 9.77 % | 76,3 | 69,4 | 0.66 % | 0.56 % | 0.51 % |
| CD109 | 178 | 122 | 19.4 % | 1.93 % | 1.93 % | 91,3 | 75,8 | 2.40 % | 2.28 % | 2.22 % |
| CD191 | 564 | 455 | 81.3 % | 8.61 % | 7.59 % | 305 | 266 | 42.1 % | 37.5 % | 37.3 % |
| CD107a | 472 | 337 | 67.9 % | 6.76 % | 6.76 % | 263 | 183 | 16.0 % | 15.3 % | 15.1 % |
| CD92 | 1185 | 1163 | 99.2 % | 9.18 % | 7.65 % | 708 | 699 | 96.5 % | 82.7 % | 77.4 % |
| HLA-A,B,C | 1067 | 988 | 99.3 % | 25.6 % | 24.9 % | 660 | 644 | 99.7 % | 95.0 % | 94.9 % |
| CD47 | 7586 | 7365 | 100 % | 51.5 % | 37.9 % | 4699 | 4903 | 100 % | 54.3 % | 51.9 % |
| CD93 | 908 | 853 | 85.8 % | 9.18 % | 8.88 % | 575 | 507 | 67.3 % | 40.3 % | 38.8 % |
| CD66a/c/e | 9686 | 10675 | 97.6 % | 8.64 % | 6.30 % | 6136 | 6015 | 100.0 % | 88.1 % | 85.5 % |
| CD97 | 2244 | 2270 | 100 % | 10.8 % | 10.4 % | 1467 | 1464 | 100 % | 58.5 % | 56.5 % |
| CD32 | 2792 | 2992 | 99.3 % | 10.1 % | 9.11 % | 1840 | 1906 | 100.0 % | 86.5 % | 82.2 % |
| CD262 | 73,9 | 70,6 | 0.60 % | 0.39 % | 0.39 % | 128 | 118 | 2.03 % | 1.10 % | 1.07 % |
| CD28 | 68,8 | 61,6 | 1.07 % | 0.68 % | 0.68 % | 125 | 123 | 0 % | 0 % | 0 % |
| CD278 | 47,9 | 42,4 | 0.75 % | 0.072 % | 0.072 % | 88,1 | 83,5 | 0.21 % | 0.20 % | 0.20 % |
| CD1a | 56,3 | 52,6 | 0.76 % | 0.071 % | 0.071 % | 105 | 92,5 | 2.71 % | 2.55 % | 2.51 % |
| CD30 | 64,9 | 62,9 | 0.12 % | 0.078 % | 0.077 % | 122 | 113 | 0 % | 0 % | 0 % |
| CD70 | 56 | 50,1 | 0.50 % | 0.049 % | 0.049 % | 113 | 99 | 2.58 % | 2.45 % | 2.41 % |
| CD3 | 64,3 | 60,4 | 0.64 % | 0.40 % | 0.40 % | 135 | 121 | 5.88 % | 0.073 % | 0.025 % |
| CD290 | 75,3 | 71,9 | 0.52 % | 0.33 % | 0.32 % | 167 | 152 | 0 % | 0 % | 0 % |
| IFN-γ R b chain | 78,1 | 60,4 | 3.44 % | 0.33 % | 0.33 % | 186 | 147 | 8.39 % | 7.94 % | 7.79 % |
| CD261 | 82 | 70,6 | 1.61 % | 1.03 % | 1.02 % | 200 | 116 | 11.1 % | 6.16 % | 5.94 % |
| CD202b | 77 | 65,5 | 1.54 % | 0.16 % | 8.09 % | 266 | 257 | 30.9 % | 27.0 % | 26.1 % |
| Cadherin 11 | 51,4 | 46,2 | 0.40 % | 0.040 % | 0.040 % | 295 | 278 | 32.7 % | 31.3 % | 30.7 % |
